# Peritumoral lymphangiogenesis induced by vascular endothelial growth factor C and D promotes lymph node metastasis in breast cancer patients

**DOI:** 10.1186/1477-7819-10-165

**Published:** 2012-08-20

**Authors:** Ying-Chun Zhao, Xiao-Jian Ni, Yong Li, Min Dai, Zhong-Xu Yuan, Yong-Yun Zhu, Chuan-Yu Luo

**Affiliations:** 1 Department of Breast Surgery, The Second People’s Hospital of Wuhu Affiliated with Wannan Medical College, 231 Jiuhuashan Road, Wuhu, 241000, China; 2 Department of General Surgery, The First Affiliated Hospital of Nanjing Medical University, 300 Guangzhou Road, Nanjing, 210029, China; 3 Department of Pathology, The Second People’s Hospital of Wuhu Affiliated with Wannan Medical College, 231 Jiuhuashan Road, Wuhu, 241000, China

**Keywords:** Breast cancer, Lymphangiogenesis, Metastasis, VEGF-C, VEGF-D

## Abstract

**Background:**

Mounting clinical and experimental data suggest that the migration of tumor cells into lymph nodes is greatly facilitated by lymphangiogenesis. Vascular endothelial growth factor (VEGF)-C and D have been identified as lymphangiogenic growth factors and play an important role in tumor lymphangiogenesis. The purpose of this study was to investigate the location of lymphangiogenesis driven by tumor-derived VEGF-C/D in breast cancer, and to determine the role of intratumoral and peritumoral lymphatic vessel density (LVD) in lymphangiogenesis in breast cancer.

**Methods:**

The expression levels of VEGF-C/D were determined by immunohistochemistry, and intratumoral LVD and peritumoral LVD were assessed using immunohistochemistry and the D2-40 antibody in 73 patients with primary breast cancer. The associations of intratumoral LVD and peritumoral LVD with VEGF-C/D expression, clinicopathological features and prognosis were assessed.

**Results:**

VEGF-C and D expression were significantly higher in breast cancer than benign disease (*P* < 0.01). VEGF-C (*P* < 0.001) and VEGF-D (*P* = 0.005) expression were significantly associated with peritumoral LVD, but not intratumoral LVD. Intratumoral LVD was associated with tumor size (*P* = 0.01). Peritumoral LVD was significantly associated with lymph node metastasis (LNM; *P* = 0.005), lymphatic vessel invasion (LVI; *P* = 0.017) and late tumor,node, metastasis (TNM) stage (*P* = 0.011). Moreover, peritumoral LVD was an independent risk factor for axillary lymph node metastasis, overall survival and disease-free survival in multivariate analysis.

**Conclusions:**

This study suggests that tumor-derived VEGF-C/D induce peritumoral lymphangiogenesis, which may be one mechanism that leads to lymphatic invasion and metastatic spread. Peritumoral LVD has potential as an independent prognostic factor in breast cancer patients.

## Background

Breast carcinoma is the most common malignant tumor in females, and the incidence of this disease has significantly increased from one per twenty women in the 1960s to one in eight today [1]. Lymph node status is the most important prognostic factor in patients with breast cancer. Lymphatic metastasis was previously thought to be a passive process, by which detached tumor cells enter lymph nodes in the vicinity of a primary tumor via pre-existing lymphatic vessels [2]. Lymphangiogenesis is the formation of new lymphatic vessels, and during the last several years, lymphangiogenesis driven by tumor-derived lymphangiogenic growth factors has been firmly established as a novel mechanism for cancer progression.

Lymphatic metastasis represents a series of sequential processes that include the dissemination and invasion of tumor cells from the primary tumor into the surrounding stromal tissue, penetration of the tumor cells across the lymphatic walls, implantation in the regional lymph nodes, and extravasation and proliferation in the parenchyma of target organs [3]. Two members of the vascular endothelial growth factor family, VEGF-C and VEGF-D, have been defined as lymphangiogenic growth factors and play an important role in tumor lymphangiogenesis via activation of the VEGF receptor (VEGFR)-3, which is expressed in lymphatic endothelial cells. In experimental tumor models, expression of VEGF-C and VEGF-D has been shown to induce lymphangiogenesis and correlate with lymphatic invasion and nodal metastasis [4,5]. Elevated expression of VEGF-C has been reported in 30 to 40 percent of breast cancers, and is associated with a higher incidence of lymphatic vessel invasion, lymph node metastasis and poorer disease-free survival (DFS) [6,7]. VEGF-D is involved in the lymphatic spread of breast cancer cells and is an independent prognostic factor for poor outcome in breast cancer [8].

The intratumoral and peritumoral lymphatic systems play distinct biological roles in the behavior and prognosis of tumors. Intratumoral lymphatic endothelial cells are capable of proliferation, suggestive of *de novo* lymphangiogenesis. A correlation between intratumoral lymphatic vessel density (LVD) and nodal metastasis has been reported in some solid tumors [9,10], but not in other studies where nonfunctional intratumoral lymphatics were quantified using dye uptake measurements [11]. Tumor-derived VEGF-C and D can induce either intratumoral or peritumoral lymphangiogenesis; however, the relative importance of the intratumoral lymphatic vessels and peritumoral lymphatic vessels in metastasis of breast cancer cells to the draining lymph nodes remains unclear.

Therefore, in the present study we quantified the intratumoral LVD and peritumoral LVD in primary invasive ductal breast carcinoma using D2-40 immunohistochemistry, and correlated these results with VEGF-C and D expression, clinicopathological characteristics and patient outcome.

## Methods

### Patients and follow-up

Paraffin-embedded specimens were obtained from 73 patients aged 29 to 75 years (mean, 53.79 ± 14.09 years) who had primary invasive ductal breast cancer and received surgical treatment between January 2005 and December 2006 at the Department of Breast Surgery, Wuhu Second People’s Hospital, Wannan Medical College, China. The study was carried out with the approval of the Research Ethics Committee of Wuhu Second People’s Hospital affiliated to Wannan Medical College. Full clinical and pathological data was collected for all 73 patients. The clinicopathologic features of the patients were summarized in Table 1. No patients had distant metastasis or received preoperative chemotherapy or radiotherapy before surgery. Benign tissues from 20 patients with mammary fibroma were selected as controls.

**Table 1:** Association of P-LVD and I-LVD with the clinicopathological features of 73 primary breast cancer patients

**Factor**	**Cases (N)**	**I- LVD (mean ± SD)**	***P*-value**	**P-LVD (mean ± SD)**	***P*-value**
Age					
>50	46	5.41 ± 2.09	0.87	8.63 ± 2.92	0.62
≤50	27	5.59 ± 1.99		9.02 ± 3.90	
Grade					
I / II	55	5.56 ± 2.05	0.58	8.17 ± 2.88	0.47
III	18	5.23 ± 2.02		8.97 ± 3.42	
Size					
≤3 cm	33	6.12 ± 1.95	0.01	8.24 ± 3.47	0.24
>3 cm	40	4.94 ± 1.98		9.22 ± 3.12	
LNM					
Negative	34	5.58 ± 1.92	0.74	7.57 ± 3.10	**0.005**
Positive	39	5.38 ± 2.15		9.82 ± 3.13	
LVI					
Negative	48	5.57 ± 2.11	0.38	8.04 ± 2.89	**0.017**
Positive	25	5.29 ± 1.96		10.19 ± 3.61	
TNM					
I - II	41	5.34 ± 2.05	0.72	7.92 ± 3.44	**0.011**
III	32	5.62 ± 2.04		9.69 ± 2.91	

I-LVD, intratumoral lymphatic vessel density; LNM, lymph node metastasis; LVI, lymphatic vessel invasion; P-LVD, peritumoral lymphatic vessel density; TNM, tumor,node,metastasis stage.

All of the breast cancer patients received post-operative adjuvant therapy consisting of combination chemotherapy and hormone treatment, and were followed up clinically for at least 5 years after surgery. The average follow-up time was 55 months (range 8 to 73 months). Follow-up examinations included a physical examination, X-ray, ultrasound exam and CT scan. Recurrence was determined by clinical and radiological examinations or histological confirmation.

### Immunohistochemical staining

Single D2-40, VEGF-C or VEGF-D immunohistochemistry was performed on formalin-fixed, paraffin-embedded sections. The sections (4 μm) were cleared, treated with 0.3% H_2_O_2_ for 10 minutes at room temperature to inhibit endogenous peroxidase activity, and then placed in 0.01 mM/L sodium citrate (pH 6.0) and heated in a microwave oven for antigen retrieval. The slides were incubated with mouse monoclonal VEGF-C primary antibody (1:100, Santa Cruz Biotechnology, Santa Cruz, CA, USA), mouse monoclonal VEGF-D primary antibody (1:100, Santa Cruz Biotechnology), or D2-40 mouse monoclonal antibody (1:25, Signet Laboratories, Dedham, MA, USA) at 4°C overnight in a humidified atmosphere, rinsed three times in 0.1 mM/L PBS for 2 minutes, incubated for 30 minutes at room temperature with goat anti-mouse horseradish peroxidase (Boster, Wuhan, China) and staining was developed using 3′3-diaminobenzidine. The primary antibodies were replaced with non-specific rabbit immunoglobulin G (IgG) to prepare the negative control slides.

The immunohistochemical staining results were interpreted by two experienced pathologists and the mean staining density was determined using ImagePro Plus 6.0 (ImagePro, Bethesda, MD, USA). VEGF-C and VEGF-D staining were semi-quantitatively assessed by combining the immunohistochemical staining intensity [none (0), weak (1), moderate (2) or strong (3)] with the percentage of tumor cells stained [0 (0%), 1 (1 to 10%), 2 (11 to 49%) or 3 (50 to 100%)]. The raw data were then converted to an Immunoreactive Score (IRS) by adding the scores for the staining intensity and percentage of tumor cells stained [12]. An IRS of 0 to 2 was considered ‘-’ (negative), 3 as ‘+’, 4 to 5 as ‘++’, 6 as ‘+++’ and 7 as ‘++++’. Consensus opinions were used to assign final IRS scores to disputed cases before data analysis.

### Assessment of LVD

Intratumoral and peritumoral LVD were determined by the hotspot method as previously described [13]. Briefly, intratumoral LVD (located at center of the tumor) and peritumoral LVD (located in the peripheral tissue within 2 mm of the tumor, adjacent to the invasive front) were assessed. In all cases, LVD was independently determined by two pathologists, who counted the number of D2-40-positive vessels in five high-power fields of view within the selected areas. Mean values were recorded for these counts. Lymphatic vessel invasion (LVI) was defined as the presence of at least one tumor cell cluster within the D2-40-positive vessels [14]. High intratumoral and peritumoral LVD were defined as LVD values higher than the respective median LVD values for all patients.

### Statistical analysis

Statistical analysis was performed using SPSS 15.0 (SPSS, Chicago, IL, USA). The Mann -Whitney *U* test or analysis of variance (ANOVA) were used to compare intratumoral and peritumoral LVD values, according to the clinicopathological variables. Multivariate analysis of risk factors for lymph node metastasis was performed using multivariate logistic regression analysis. Overall survival (OS) and disease-free survival (DFS) curves were plotted using the Kaplan - Meier method and compared using the log-rank test. A multivariate model was generated using Cox stepwise regression analysis and used to evaluate the significance of the independent associations between the covariates and DFS and/or OS. All statistical tests were two sided and significance was defined as *P* < 0.05.

## Results

### VEGF-C and VEGF-D expression in human breast cancer

**Figure 1:**
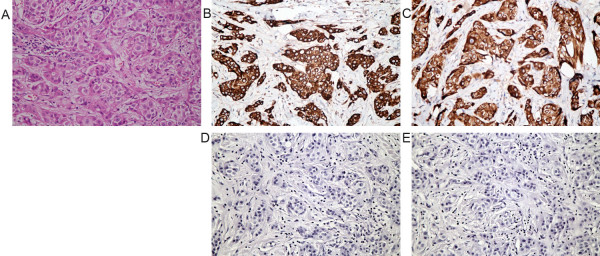
**Immunohistochemical analysis of VEGF-C and VEGF-D expression in primary breast carcinoma. **(**A**) Histopathology by hematoxylin-eosin staining of breast cancer; (**B**) Strong expression of VEGF-C and (**D**) negative control in breast cancer; (**C**) Strong expression of VEGF-D and (**E**) negative control in breast cancer. Diffuse, strong positive VEGF-C (**B**) and VEGF-D (**C**) immunostaining was mainly observed in the cytoplasm of breast cancer cells (magnification × 400). VEGF-C/D, vascular endothelial growth factor C /D.

VEGF-C and VEGF-D immunoreactivity were both observed as positive cytoplasmic staining in breast cancer cells (Figure 1). VEGF-C expression was not detected in the breast cancer tissues of 11/73 patients (15.1%); 17/73 (23.3%) of the patients were ‘+’, 26/73 (35.6%) were ‘++’, 12/73 (16.4%) were ‘+++’ and 7/73 (9.6%) were ‘++++’ for VEGF-C. VEGF-D expression was not detected in 18/73 patients (24.7%); 20/73 (27.4%) of the patients were ‘+’, 21/73 (28.8%) were ‘++’, 9/73 (12.3%) were ‘+++’ and 5/73 (6.8%) were ‘++++’ for VEGF-D. Weak VEGF-C and D immunoreactivity was observed in 5/20 (25%) and 3/20 (15%) of the mammary fibroma samples, respectively; the remainder of the control samples did not express VEGF-C/D. The expression levels of VEGF-C and D were significantly higher in primary breast carcinoma than the control fibroma tissues (*P* < 0.01; Table 2.)

**Table 2:** Association of VEGF-C and VEGF-D expression with P-LVD and I-LVD in breast cancer

		**Cases (N)**	**I- LVD (mean ± SD)**	***P*-value**^**a**^	**P-LVD (mean ± SD)**	***P*-value**^**a**^
VEGF-C	Negative	11	5.17 ± 2.23	0.96	5.77 ± 2.47	**<0.001**
	+	17	5.72 ± 2.02		7.74 ± 2.75	
	++	26	5.41 ± 1.94		9.03 ± 2.88	
	+++	12	5.46 ± 2.19		10.82 ± 3.12	
	++++	7	5.59 ± 2.44		11.53 ± 3.26	
VEGF-D	Negative	18	5.19 ± 2.25	0.88	6.78 ± 2.86	**0.005**
	+	20	5.35 ± 1.81		8.28 ± 2.61	
	++	21	5.53 ± 1.86		9.96 ± 3.42	
	+++	9	5.81 ± 2.53		10.13 ± 3.36	
	++++	5	6.16 ± 2.47		11.66 ± 3.06	

I-LVD, intratumoral lymphatic vessel density; P-LVD, peritumoral lymphatic vessel density; VEGF-C/D, vascular endothelial growth factor C /D; ^a^One-way analysis of variance.

### Characteristics of intratumoral lymphatics and peritumoral lymphatics in breast cancer

**Figure 2:**
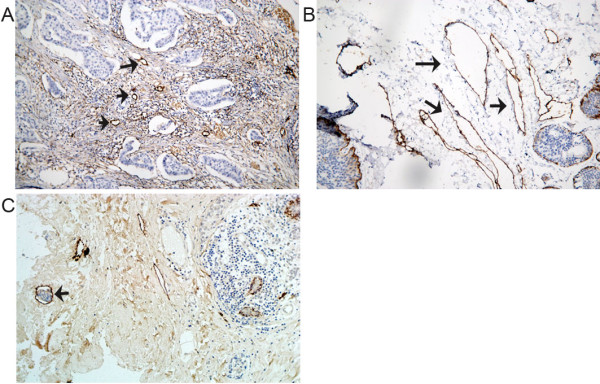
**Immunohistochemical analysis of lymphatic vessels in primary breast carcinoma. **(**A**) Intense, specific D2-40 immunoreactivity was only observed in lymphatic endothelial cells. The intratumoral lymphatic vessels are small, irregular and collapsed (arrow). (**B**) The peritumoral lymphatic vessels located at the invasive edge of tumors are frequent, often large and dilated (arrow); magnification × 200.(**C**) Immunohistochemical visualization of invading breast cancer cells in the lymphatic vessels of the peritumoral region of a primary breast carcinoma. The black arrow indicates lymphatic vessel invasion (LVI); magnification × 200.

D2-40-stained lymphatic vessels were unevenly distributed throughout the breast tumors. Lymph vessels within the tumor mass were generally small, irregular and collapsed (Figure 2A). The lymph vessels in the peritumoral areas were more frequent, larger and dilated (Figure 2B). As expected, D2-40 immunostaining highlighted the presence of lymphatic invasion (Figure 2C), which is usually present at the periphery of tumors. No significant difference was observed between the intratumoral LVD of breast carcinoma and the LVD of control tissues (5.47 ±2.03 vs. 5.25 ± 1.73, *P* > 0.05). However, the peritumoral LVD (8.77 ± 3.30) was significantly higher than the intratumoral LVD and LVD of control tissues (*P* < 0.05).

### Relationship of intratumoral LVD and peritumoral LVD with VEGF-C/D expression and clinicopathological features

Significant correlations were observed between the expression of VEGF-C/D and peritumoral LVD in primary breast carcinoma. Specifically, peritumoral LVD increased as VEGF-C/D expression increased (*P* < 0.01). No similar relationships between VEGF-C/D and intratumoral LVD were observed (Table 2).

The association of intratumoral LVD and peritumoral LVD with the clinicopathological features of breast cancer are shown in Table 1. Intratumoral LVD and peritumoral LVD did not correlate with patient age or tumor grade; however, intratumoral LVD correlated with tumor size (*P* = 0.01) and peritumoral LVD correlated significantly with lymph node metastasis (*P* = 0.005), LVI (*P* = 0.017) and TNM stage (*P* = 0.011; Table. 1).

### Predictive value of intratumoral LVD and peritumoral LVD for axillary lymph node metastasis

Multivariate logistic regression analysis indicated that VEGF-C expression, peritumoral LVD and the presence of LVI were significantly associated with axillary lymph node metastasis (*P* = 0.027, *P* = 0.006 and *P* = 0.019, respectively). Intratumoral LVD and VEGF-D expression had no predictive value for axillary lymph node metastasis in breast cancer (Table 3).

**Table 3:** Multivariate logistic regression analysis of the factors affecting axillary lymph node metastasis in primary breast carcinoma

	***P*-value**	**Odds ratio**	**95% CI**
P-LVD	**0.006**	2.255	1.269-4.008
I-LVD	0.168	0.709	0.434-1.156
VEGF-C	**0.027**	11.837	1.327-45.584
VEGF-D	0.147	0.249	0.038-1.627
LVI	**0.019**	4.167	2.755-15.363

CI, confidence interval; I-LVD, intratumoral lymphatic vessel density; LVI, lymphatic vessel invasion; P-LVD, peritumoral lymphatic vessel density; VEGF-C/D, vascular endothelial growth factor C /D.

### Survival analysis

**Figure 3:**
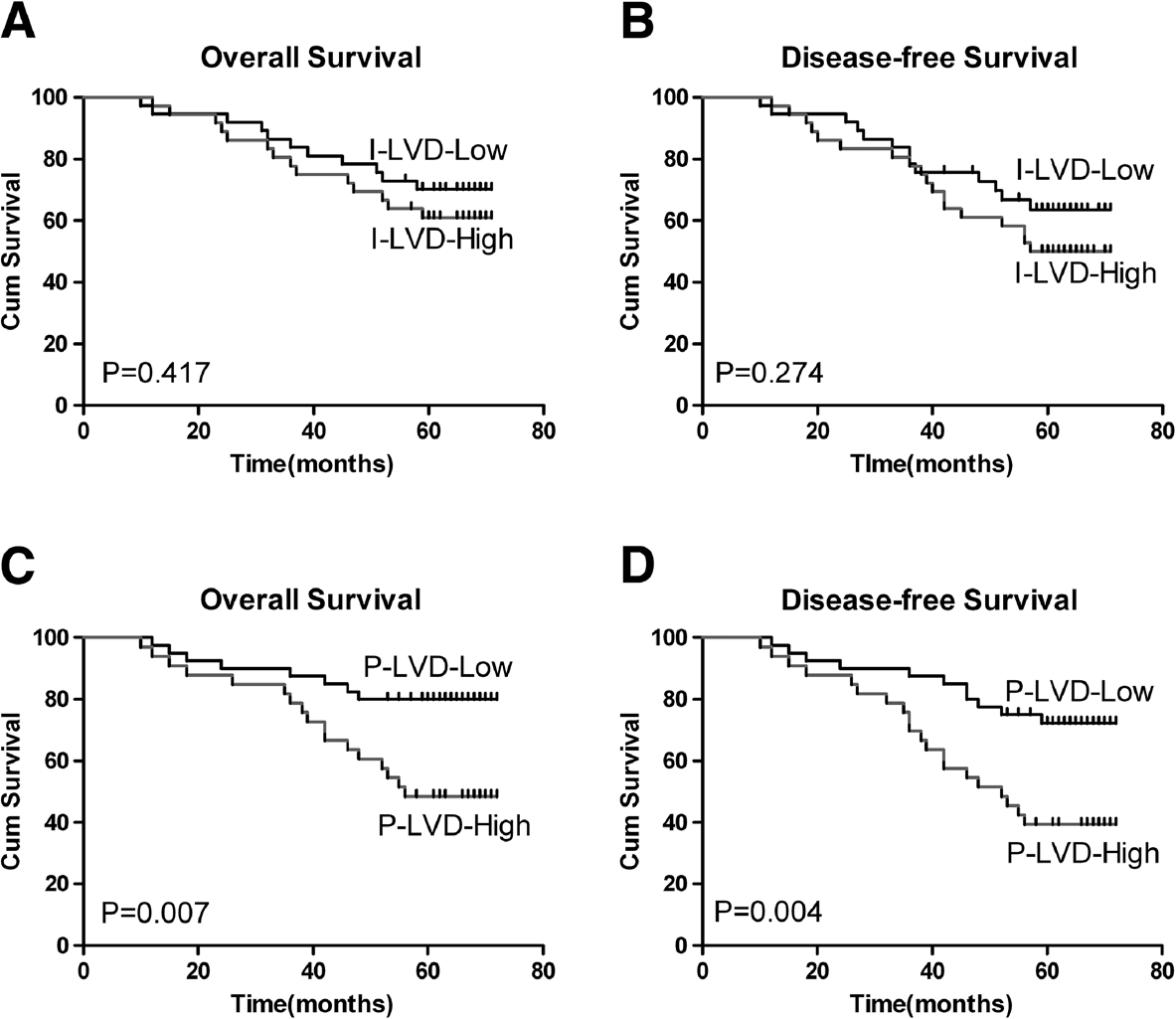
**Kaplan-Meier disease-related overall survival (A and C) and disease-free survival (B and D) curves; stratified by low versus high intratumoral LVD (A and B) and low versus high peritumoral LVD (C and D) in invasive ductal breast carcinoma. ** The median LVD values were used as cutoff values. LVD, lymphatic vessel density.

The 5-year DFS rate for the 73 patients was 57.53% (42/73), and the 5-year OS rate was 65.75% (48/73). We divided the 73 patients into two groups, using the median intratumoral LVD and peritumoral LVD values (6.0 and 9.0, respectively) as cut-off points. In univariate survival analysis, intratumoral LVD demonstrated a non-significant trend towards OS (*P* = 0.417; Figure 3A) and DFS (*P* = 0.274; Figure 3B). However, high peritumoral LVD was significantly associated with poorer OS (*P* = 0.007; Figure 3C) and DFS (*P* = 0.004; Figure 3D). Furthermore, multivariate regression analysis indicated that peritumoral LVD was an independent prognostic factor for both OS (*P* <0.001) and DFS (*P* = 0.001). Moreover, the presence of LNM and TNM stage also served as independent predictors for both OS and DFS (LNM: *P* = 0.037 and 0.040; TNM stage, *P* = 0.035 and 0.006, respectively). However, no significant correlations were observed between intratumoral LVD, VEGF-C, VEGF-D or LVI and any survival outcome (Table 4).

**Table 4:** Cox regression analysis of the independent factors affecting overall survival and disease-free survival in primary breast carcinoma

	**Overall survival**		**Disease-free survival**	
	**OR (95% CI)**	***P*-value**	**OR (95% CI)**	***P*-value**
P-LVD	3.077 (2.086-4.539)	**0.000**	2.245 (1.602-3.144)	**0.001**
I-LVD	0.927 (0.709-1.212)	0.579	1.086 (0.830-1.422)	0.547
VEGF-C	0.637 (0.225-1.804)	0.396	0.432 (0.169-1.103)	0.079
VEGF-D	1.900 (0.690-5.230)	0.214	2.025 (0.842-4.872)	0.115
LNM	3.564 (1.081-11.749)	**0.037**	13.333 (1.125-68.667)	**0.040**
LVI	1.218 (0.457-3.250)	0.693	1.421 (0.566-3.567)	0.454
TNM stage	4.464 (1.111-18.182)	**0.035**	5.917 (1.681-20.833)	**0.006**

I-LVD, intratumoral lymphatic vessel density; LNM, lymph node metastasis; LVI, lymphatic vessel invasion; P-LVD, peritumoral lymphatic vessel density; TNM, tumor,node,metastasis stage.

## Discussion

Metastatic spread of tumor cells is responsible for the majority of cancer-related deaths. In similar manner to other carcinomas, breast cancer has a predilection to initially metastasize to the regional lymph nodes [15], most commonly via the lymphatic system [16]. Lymphangiogenesis is considered to be a key process during lymphatic metastasis [2]; however, the role of lymphangiogenesis in promoting the metastatic spread of tumor cells via lymphatic vessels has received little attention in the last decade. This has been in part due to the difficulty of studying lymphatic vessels, due to their morphology, and a lack of lymphatic-specific markers [17]. Recently, the D2-40 antibody has been shown to specifically recognize the glomerular podocyte membrane protein Podoplanin, and is a very sensitive and specific marker for lymphatic endothelium in most tissues, especially breast cancer [18]. In ‘The First International Consensus on The Methodology of Lymphangiogenesis Quantification in Solid Human Tumors’ [19], Podoplanin was considered to be the most reliable marker of lymphatic vessels currently available. In this study, D2-40 produced strong and specific lymphatic vessel immunoreactivity in breast cancer. In agreement with previous reports [20], the D2-40-positive lymphatic vessels observed in this study usually had an irregular shape and thin-walled lumen devoid of red blood cells. The intratumoral vessels were generally collapsed, whereas the peritumoral vessels were wider, with more dilated lumina, and tumor emboli were more frequently observed within the peritumoral lymphatics.

This study aimed to clarify the location of VEGF-C/D-induced lymphangiogenesis and investigate the role of intratumoral and peritumoral lymphatic vessels in lymph node metastasis and the outcome of patients with breast cancer. Lymphangiogenesis is controlled by a complex network of growth factors, cytokines and chemokines, and actively contributes to tumor metastasis [21]. Skobe *et al.* first demonstrated that VEGF-C induced lymphangiogenesis and promoted metastasis in animal studies [22]. Over-expression of VEGF-C triggers lymphangiogenesis and is associated with a higher risk of cervical lymph node metastasis in oral squamous cell carcinoma [23]. Stacker *et al.* described the induction of lymphangiogenesis by VEGF-D-over-expressing tumor cells in the skin of severe combined immunodeficient (SCID) mice [24]. Similar to a previous report [6], our results demonstrated that both VEGF-C and VEGF-D are expressed at significantly higher levels in breast cancer than in benign mammary lesions. However, previous studies have not evaluated the relationship between LVD and VEGF-C/D in different regions of tumors. We observed that peritumoral LVD was significantly higher than intratumoral LVD and the LVD of control tissues. Additionally, the peritumoral LVD was closely related to expression of VEGF-C and VEGF-D; however, intratumoral LVD was not related to VEGF-C/D. These results suggest that tumor-derived VEGF-C/D induce lymphangiogenesis around tumors, but not within breast tumors.

Considerable debate remains regarding the role of intratumoral versus peritumoral lymphatic vessels in the pathology of primary human tumors. Proliferating intratumoral lymphatics have been observed in tumor xenotransplants and in slowly growing, chemically-induced, orthotopic squamous cell carcinomas (SCC) in mice, and also in primary human skin malignant melanomas that metastasized to the lymph nodes [25,26]. In recent years, several studies have associated intratumoral LVD with tumor lymph node metastasis in pancreatic endocrine tumors [27], head and neck squamous cell carcinoma [9] and papillary thyroid carcinoma [28]. Moreover, Skobe *et al.* also suggested that over-expression of VEGF-C in breast cancer cells potently increased intratumoral lymphangiogenesis, which significantly enhanced metastasis to the regional lymph nodes and lungs [22]. However, using an experimental system, Padera *et al.* demonstrated the occurrence of metastatic spread in the absence of detectable intratumoral lymphatic vessels, and proposed that the functional lymphatics at the tumor margin are sufficient for the promotion of metastasis, as they offer a large area for tumor cell escape [11]. A recent study in an experimental model of prostate cancer also observed efficient metastasis to the lymph nodes in the absence of intratumoral lymphatics [29]. Bono *et al.* reported that peritumoral lymphatics were far more frequent than intratumoral vessels, and the extent of peritumoral lymphatic vessels correlated with nodal metastasis [30]. Most of the available data indicates a strong correlation between peritumoral lymphangiogenesis and tumor aggressiveness. Our results are in agreement with the latter studies; we observed that the density of lymphatic vessels was usually greater at the tumor periphery than intratumorally, and that a high peritumoral LVD, not intratumoral LVD, was associated with more aggressive behavior in breast carcinoma.

We also investigated the relationship between intratumoral LVD, peritumoral LVD and clinicopathological features in breast cancer. Intratumoral LVD was significantly related to the primary tumor size, with larger tumors having a lower intratumoral LVD. One possibility for this observation is that the tumor tissue lymphatics are destroyed by invading tumor cells or high interstitial fluid pressure due to the expanding tumor mass [3]. More importantly, there was a significant correlation between peritumoral LVD and lymphatic vessel invasion, lymph node metastasis and TNM clinical stage, indicating that VEGF-C/D-induced peritumoral lymphangiogenesis leads to lymphatic invasion and lymph node metastasis. The contradictory results on the role of intratumoral-lymphatic vessels and peritumoral-lymphatic vessels in tumors reflect the fact that tumor lymphangiogenesis and lymphatic metastasis are complex mechanisms, which can differ significantly in different tumor types or in tumors at different anatomic locations [31].

It was also important to examine whether intratumoral LVD or peritumoral LVD had any prognostic value in breast carcinoma. Peritumoral LVD and intratumoral LVD are risk factors for lymph node metastasis in early gastric cancer [32]. In this study, multivariate analysis indicated that only peritumoral LVD was an independent predictor of axillary lymph node metastasis. No correlations were observed between intratumoral LVD and patient outcome; however, increased peritumoral LVD was associated with poorer DFS and OS. Multivariate analysis also indicated that increased peritumoral LVD was a prognostic factor for DFS and OS in breast cancer. These findings are in agreement with other studies, as the LVD in the periphery of tumors correlates with poorer outcomes in lung [33], colorectal [34] and prostate cancer [35]. This evidence demonstrates that peritumoral lymphangiogenesis plays an important role in lymphatic metastasis and tumor progression, and that peritumoral lymphangiogenesis is an independent predictor of lymph node metastasis and prognostic factor in breast carcinoma. Most solid tumors metastasize via lymphatic invasion; therefore LNM is an important prognostic factor [3], and as expected, LNM and TNM were also prognostic factors for DFS and OS in breast cancer.

In this study, lymphatic vessel invasion (LVI) of the tumor was not associated with either overall survival rates or disease-free survival in multivariate analysis, but was weakly correlated with axillary LNM. This discrepancy could be explained in several ways. Firstly, the histopathological detection of LVI may have under-represented the true number of involved lymphatic vessels after the selection of tumor tissue regions. Secondly, the involvement of lymphatic vessels by tumor does not necessarily mean that lymph nodes have been affected, which may result in some of patients classified as having LVI, but with a lower TNM score than those with proven lymph node involvement. Consequently, LVI could be perceived as a less accurate marker of prognosis than lymph node involvement and TNM stage, which is borne out in our results, as it does not reflect the degree of distant spread. The latter may be dependent on the ability of tumor cells to migrate, and would therefore mean that LVI was not a direct marker of survival. It is however an interesting parameter that should be taken into account in the grading of tumors.

In summary, our results suggest that high levels of VEGF-C/D expression by breast tumor cells may induce lymphangiogenesis in the peritumoral region and contribute to a high peritumoral LVD, leading to increased aggressiveness, lymphatic invasion, metastatic spread and a poorer prognosis. Inhibiting the expression, or blocking the function, of VEGF-C/D to control peritumoral lymphangiogenesis is expected to lead to the development of novel therapeutic strategies for the treatment and management of breast cancer; however, further characterization of the molecular mechanisms which regulate lymphangiogenesis is still required.

## Conclusions

The present study demonstrated that VEGF-C/D expression were significantly higher in breast cancer. The expression of these factors was significantly associated with peritumoral LVD, but not intratumoral LVD. And peritumoral LVD was significantly associated with lymph node metastasis, lymphatic vessel invasion and late TNM stage. However no such relationship was found with intratumoral LVD. Moreover, peritumoral LVD was an independent risk factor for axillary lymph node metastasis, overall survival and disease-free survival. Inhibiting the expression of VEGF-C/D to control peritumoral lymphangiogenesis is expected to lead to the development of novel therapeutic strategies for breast cancer.

## Abbreviations

DFS: disease-free survival; IgG: immunoglobulin G; I-LVD: intratumoral lymphatic vessel density; IRS: immunoreactive score; LNM: lymph node metastasis; LVI: lymphatic vessel invasion; OS: overall survival; P-LVD: peritumoral lymphatic vessel density; VEGF-C/D: vascular endothelial growth factor C /D; TNM: tumor,node,metastasis stage.

## Competing interests

The authors declare that they have no competing of interests.

## Authors’ contributions

YCZ, XN and CL designed this study and carried out immnunohistochemistry staining, performed the statistical analysis, collected clinical information and drafted the manuscript. YL, MD, ZY, YYZ participated in immunohistochemistry staining, patient follow up and the statistical analysis. All authors read and approved the final manuscript.
